# Facts and Fallacies in Gastro-Intestinal Work

**Published:** 1905-02

**Authors:** L. Amster

**Affiliations:** Atlanta, Ga.


ATLANTA

### Journal-Record of Medicine.

**Successor to Atlanta Medical and Surgical Journal, Established 1855, and Southern Medical Record, Established 1870.**

OWNED BY THE ATLANTA MEDICAL JOURNAL CO.

**Published Monthly.**

VOL. VI. FEBRUARY, 1905. No. 11.

BERNARD WOLFF, M.D., EDITOR, Nos. 319–20 Prudential.

M. B. HUTCHINS, M.D., BUSINESS MANAGER, 1007-1008 Century Bldg.

J. N. LECONTE, M.D., ASSOCIATE EDITOR.

### *ORIGINAL COMMUNICATIONS.*

### FACTS AND FALLACIES IN GASTRO-INTESTINAL WORK.

BY L. AMSTER, M.D.,
ATLANTA, GA.

Gastro-enterology has of late years developed into an important specialty. Diseases of the gastro-intestinal tract form an integral part of the practice of medicine. The general practitioner, with his large and varied field, can not always devote to this particular class of diseases the attention of a specialist.

Still, in justice to himself and suffering humanity, there are salient facts in gastro-intestinal work which he can not afford to overlook.

With this fact in view, and considering that I am addressing a society largely composed of general practitioners, I have endeavored to select a subject, or rather a variety of subjects, which I trust will be interesting to you and elicit discussion.

First, I wish to state in a spirit of guilty retrospection, having been engaged in general work for seventeen years, that as a rule, the general practitioner does not devote sufficient time and attention to diagnosing and treating his stomach and intestinal cases.

A little calomel, phosphate of soda, essence of pepsin with the diagnosis of dyspepsia, and the patient is sent on his way rejoicing, shortly to find out that his ills and ails are not alleviated.

What is the cause of this indifference on the part of the physician, considering that such a large percentage of people are afflicted with stomach and bowel troubles?

If not the love of science, his material welfare would dictate a closer devotion to these interesting subjects.

To my mind, it is the lack of teaching on the part of our medical colleges. Take as an example our own Southern institutions. I have informed myself as to the advantages the students get in this work and I say "precious little."

The most brilliant array of surgeons devote a large part of the student's time to teaching him surgery, gastro-intestinal surgery, and no facility is given the student to diagnose and differentiate, when to apply and how to apply this surgery intelligently.

I ask you, gentlemen, are not the majority of students destined for general practice, and will they not meet with stomach cases almost daily in their future career? Then why do medical colleges give so little attention to a branch of medicine the knowledge of which is so useful?

And I say without fear of contradiction that just so long as medical schools persist in ignoring this fact, just so long will people suffer and die from ulcer and cancer of the gastro-intestinal tract, who could be saved if our students were taught how to diagnose these cases and administer surgical aid in time. After these general remarks, I will speak of gastro-intestinal work in detail and a few of its different facts and fallacies.

### THE TONGUE.

From time immemorial it has been held that the appearance of the tongue is an indication of the condition of the stomach.

Some people have a mania for examining their tongues; they will insist that they are bilious as long as their tongues remain coated. Of late Mathieu, Roux and Boas have controverted this theory. In order to make a practical test, Boas instructed his assistants and pupils to watch each patient's tongue and to determine what relation a furred or coated tongue bore to the different gastro-intestinal conditions. The conclusion reached was that, for diagnostic purposes, the condition of the tongue is overestimated. They found that people suffering from the most serious organic diseases had clean tongues, while patients with hardly any or no gastro-intestinal disturbance had often the worst-looking tongues.

A coated tongue may indicate intestinal infection or intoxication, as in typhoid fever or ptomaine poisoning, and it may be met in cases of malnutrition, but it certainly is not a mirror of gastro-intestinal derangement in all cases. It may be true of acute, but not of chronic conditions.

The white-coated tongue is due to a superficial desquamative dermatitis and in chronic cases it can be controlled by thorough cleansing and disinfection.

If people would pay as much attention to the toilet of their tongues as they do to their teeth, there would be fewer complaints of bad and bilious tastes and fewer coated tongues. The scraping of the tongue with a whalebone and the use of a little permanganate of patash solution will be productive of more good than all the purgatives used for the purpose of obtaining a clean tongue.

### ESOPHAGUS.

Diseases of the esophagus are often overlooked and mistaken for stomach cases. Dilatation diverticles and cancer of the esophagus, though fortunately rare, should not be overlooked by the general practitioner. Much can be done for these cases if diagnosed in time. Skiagraphy and esophagoscopy should be employed early. Bougies and oil instillations in traumatic stenoses often give excellent results, and cocaine instillations with the esophagial syringe alleviate much pain and discomfort in inoperable cases.

### DILATION AND STENOSIS OF THE PYLORUS.

The diagnosis of dilation of the stomach is often made after the most superficial examination. Many cases are taken for ectasy because the gastric percussion sound reaches the umbilicus or lower.

We must remember that the position of the stomach is not the same in every case. Some stomachs have been found in a vertical position. In gastroptosis the greater curvature is naturally dragged down and by mapping out the lesser curvature it can readily be seen that the organ is not enlarged. In some cases it is strikingly small.

In order to avoid errors it is well to remember the following conditions:

1st. *Megalogastry.*—A naturally large stomach without motor insufficiency; this is simply an abnormal condition without any clinical significance.

2d. *Gastroectasy.*—Enlargement of the stomach with motor insufficiency and its accompanying typical symptoms of stagnation, etc.

3d. *Gastroptosis.*—Total and partial. A sagging of the organ.

I have met quite a number of patients who have been told by their physicians that they were suffering from pyloric stenosis when they had neither stagnation of stomach contents nor vomiting.

If we bear in mind the fact that stagnation and vomiting are the two cardinal symptoms of pyloric stenosis we will obviate such errors in diagnosis.

Boas claims that eructations of sulphide of hydrogen are always significant of pyloric stenosis of benign character.

### ULCER AND CANCER.

Ulcer of the stomach, where hematemesis has not taken place, and ulcer of the duodenum are frequently overlooked by the general practitioner. When we see, for instance, a chlorotic young girl who gives a history of pain a half to two hours after meals, and who tells us the pain is more severe after the ingestion of coarse than after soft food, or drinking milk with the typical point of pressure usually found in gastric ulcer, we ought to pause before making a diagnosis of nervous dyspepsia.

Ulcer of the stomach is not a rare disease by any means.

Treating these cases ambulatory fashion is a mistake. We should insist upon the ideal ulcer cure as first carried out by Leubé. Rest in bed, hot poultices, rectal feeding, milk diet, etc.

In connection with the milk treatment employed in cases of gastric ulcer, I wish to say that some patients rebel against the exclusive milk diet. Boas has succeeded in administering the milk cure to patients who have not done well before, by simply ordering them to cleanse their mouths thoroughly after each glass of milk.

The consensus of opinion of the foremost American and foreign authorities is that cancer is on the increase—so much so that it bids fair to rival tuberculosis. The study of cancer deserves our most serious consideration. If we fail to diagnose a case of cancer early, it is a mistake to console ourselves by thinking that, considering its hopelessness, there is not much harm done. On the contrary, if it is recognized in time we can do much, medically and surgically, to cure and prolong life.

In the large majority of cases, the history of the patient should make us suspicious of cancer. Unless the cancer develops upon the basis of an old ulcer, we shall usually find that the patient will tell us that he has never had any trouble with his digestion, that he noticed the first symptoms recently, that he has lost considerably in weight, and that he has lost his appetite and has a particular dislike for meats. These symptoms, together with the age of the patient, should arouse our suspicions and make us investigate the case more carefully. To wait until cachexia, the characteristic vomit and symptoms of pyloric obstruction appear does not give the patient a chance for life.

Dr. Mayo, of Rochester, Minn., the eminent gastrointestinal surgeon, at a recent meeting, urged the profession to do more exploratory operations, in order to clear up the diagnosis of cancer of the gastro-intestinal tract. This may be all right in theory; not in practice. It may easily be accomplished in hospital, but not in private work.

We know that unless the motive power of the stomach is disturbed, as in cancer of the pylorus, the cases are not urgent. In cancer of the lesser curvature or of the fundus, digestion and nutrition are not very materially interfered with and under these conditions very few patients will submit to exploratory operations.

With the history of the case, with the chemical and microscopic aids we have for diagnosing cancer and, in addition, the test of what Boas calls "occult hemorrhage," and with the appearance of supraclavicular glands, cancer of the stomach can be diagnosed in most cases without the presence of a tumor.

The greatest obstacle to an early diagnosis is the fact that many cases do not present themselves sufficiently early.

As stated before, unless the motility of the stomach is interfered with, as in cancer of, or near, the pylorus, the symptoms are not urgent enough for the patient to consult a physician.

### CHOLELITHIASIS.

Numerous mistakes are made, cholelithiasis being treated for stomach trouble. A close investigation in these cases will reveal the fact that the attacks of pain are independent of the taking of food. A large majority of patients sueffring from cholelithiasis seem to be women who have borne children. Boas calls attention to special points of pressure, front and back, found ordinarily in cholelithiasis.

Cases with enlargement of the liver, jaundice, and attacks of pain are readily made out; so are cases without jaundice, but with enlargement of the liver and pain. Most errors occur in simple cases without jaundice and without enlargement of the liver. These are the cases that can be benefited by the physician. Medical treatment, with rest in bed, hot poultices, Carlsbad salt, and diet, yield excellent results.

### HERNIA OF THE LINEA ALBA.

Small ruptures of the linea alba often give rise to digestive disturbances. A close inspection and palpation of the abdomen will readily reveal this condition, which is best treated surgically.

### DIET AND MEDICINE.

The successful treatment of gastro-intestinal diseases depends very largely, if not, exclusively, upon diet. It is necessary that we thoroughly understand the *modus operandi* of digestion. The result of investigations made by Leube, Penzoldt, Pawlow, and others, with various foods, upon healthy and diseased stomachs and intestines, aids us materially in the intelligent application of diet.

It is important for us to know when food is most digestible. Hemmeter gives the following definition: "One that makes relatively small demands upon the secretory and motor functions of the stomach, which is readily absorbed and produces no subjective complaint or feeling of discomfort." The practice of handing to patients stereotyped diet lists, prepared by advertising drug firms, can not be too strongly condemned. The diet should be written out for each patient according to the indications of the case.

The quantity of food should also be considered. Rest to an abused and overworked stomach and plenty of food and work for a stomach that has been made to forget its mission are far more essential than medicines.

The folly of indiscriminately using lavage is so obvious that it does not admit of discussion.

Drugs in the treatment of gastro-intestinal diseases are more adjuvant and palliative, than curative. The employment of the numerous preparations of pepsin is useless. Pepsin is indicated only in the lowering of the secretion and atrophy of the peptic glands, as in the secondary gastritis due to carcinoma. If there is any virtue in these preparations, it is due to the alcohol in the elixirs or to psychic effect. Of late two preparations—animal gastric juices—have supplanted pepsin, *i. e.* gasterine, from the dog, and dyspeptine from the hog. French and German clinicians report good results from their employment.

As to the employment of hydrochloric acid in sub. and anacidity, there is a doubt if the small doses that can be safely given are sufficient to supply the want of the acid necessary for digestion. At best HCl. seems to act more as an appetizer and a stimulus to the secretion of gastric juice.

The effect of alkalies in hyperacidity is transient and the administration of food that binds the hydrochloric acid in the stomach serves a better purpose.

Pepsin and bicarbonate of soda in combination are still often prescribed. This is an absurdity which ought not to be committed even by the least intelligent.

Time and the extent of the subject will not permit me to discuss the intestinal phase of the question. I would like to call your attention, however, to a great evil that is universally prevalent, namely, the inordinate abuse of cathartics. Biliousness, that dumping ground of ignorance, seems to hold unrestricted sway over our livers or imaginations. Physicians, for the want of a better diagnosis, seem to encourage the laity in this fallacy. The result is that the gullible public is purged "ad nauseam" by those liberally advertised medicines. No wonder that the intestines, instead of being coaxed by judicious diet and treatment, are whipped into obedience and worn out by the extraordiuary and unnatural work they are made to perform, until complete exhaustion sets in.

In the realm of intestinal diseases there is no condition so satisfactory to treat as that of habitual constipation. Diet alone will often accomplish the most marvelous results in the most aggravated cases. The additions of warm oil instillations, massage and electricity to a judicious diet are far more effective than drugs.

In treating habitual constipation we must not lose sight of the fact that there is an "atonic" and a "spastic" variety, each requiring different treatment. Ball and bandlike feces are the results of spasm of the intestines and not of stenosis as supposed by some.

Before concluding I wish to emphasize the fact that the American family physician has a mission to perform by demonstrating to his patients the fallacy of a life of excess and its inevitable baneful consequences upon the digestive apparatus and nervous system. The unrest of the strenuous American life, the irrational practice of filling one's stomach with rich food, steaming hot breads and biscuits; the inordinate use of icewater and strong alcoholics, sedatives and narcotics, are subjects worthy of discussion between physician and patient. Carlsbad, Kissingen, and the multitude of European sanitaria, for the treatment of the digestive organs, are filled with the wrecks caused by the folly of American life. The number of neurasthenics and dyspeptics can be lessened by the dissemination of knowledge of how we ought to live?

